# Cellular Localization and Processing of Primary Transcripts of Exonic MicroRNAs

**DOI:** 10.1371/journal.pone.0076647

**Published:** 2013-09-20

**Authors:** Izabella Slezak-Prochazka, Joost Kluiver, Debora de Jong, Gertrud Kortman, Nancy Halsema, Sibrand Poppema, Bart-Jan Kroesen, Anke van den Berg

**Affiliations:** Department of Pathology and Medical Biology, University Medical Center Groningen, University of Groningen, Groningen, The Netherlands; Colorado State University, United States of America

## Abstract

Processing of miRNAs occurs simultaneous with the transcription and splicing of their primary transcripts. For the small subset of exonic miRNAs it is unclear if the unspliced and/or spliced transcripts are used for miRNA biogenesis. We assessed endogenous levels and cellular location of primary transcripts of three exonic miRNAs. The ratio between unspliced and spliced transcripts varied markedly, i.e. >1 for *BIC*, <1 for pri-miR-146a and variable for pri-miR-22. Endogenous unspliced transcripts were located almost exclusively in the nucleus and thus available for miRNA processing for all three miRNAs. Endogenous spliced pri-miRNA transcripts were present both in the nucleus and in the cytoplasm and thus only partly available for miRNA processing. Overexpression of constructs containing the 5’ upstream exonic or intronic sequence flanking pre-miR-155 resulted in strongly enhanced miR-155 levels, indicating that the flanking sequence does not affect processing efficiency. Exogenously overexpressed full-length spliced *BIC* transcripts were present both in the nucleus and in the cytoplasm, were bound by the Microprocessor complex and resulted in enhanced miR-155 levels. We conclude that both unspliced and spliced transcripts of exonic miRNAs can be used for pre-miRNA cleavage. Splicing and cytoplasmic transport of spliced transcripts may present a mechanism to regulate levels of exonic microRNAs.

## Introduction

MicroRNAs (miRNAs) are small (~22nt) noncoding RNA molecules that negatively regulate gene expression by binding to the 3’ untranslated region (3’ UTR) of their target mRNAs [1]. MiRNAs play an important role in cellular processes like apoptosis, proliferation and differentiation. Altered miRNA expression profiles have been associated with various diseases including most, if not all, types of cancer [2]. This suggests that regulation of miRNA levels is important for normal cellular functioning. Regulation of the miRNA levels may include any of the regulatory mechanisms involved in normal gene expression, such as transcriptional or epigenetic control of transcription. In addition to these transcriptional regulations, miRNA levels can also be regulated at the post-transcriptional level during miRNA processing (reviewed in 3).

The first step in miRNA processing, i.e. cleavage of the primary miRNA transcript (pri-miRNA) by the Drosha/DGCR8 complex, is restricted to the nucleus [4-7]. The miRNA stem-loop structures can be located in introns of protein-coding or noncoding RNA genes, in exons of noncoding genes or in intergenic regions [8]. The vast majority of the human miRNAs are located in introns. Approximately 10% of the miRNAs, including miR-155, miR-146a, miR-22, miR-137, miR-34c and let-7b, reside within exons of noncoding genes [9-11]. Current knowledge about processing of pri-miRNAs has been obtained mainly for intronic or intergenic miRNAs [10,12-16]. Processing of intronic miRNAs occurs co-transcriptionally in cooperation with splicing of the primary transcript [10,12,13,15,16]. The Microprocessor complex and the spliceosome are associated in one complex, and co-produce precursor miRNAs (pre-miRNA) and spliced transcripts from the unspliced pri-mRNA [15]. Splicing is not required for pri-miRNA processing [10], but spliceosome assembly may promote release of the pre-miRNA from introns of pri-miRNA [15]. For exonic miRNAs, pre-miRNA release will disrupt the exon of the pri-miRNA and affect formation of spliced transcripts. Therefore, it is more likely that unspliced pri-miRNA transcripts of exonic miRNAs produce either pre-miRNAs or spliced transcripts. The processing of exonic miRNAs has not yet been studied in detail.

One well-known exonic miRNA, miR-155, is processed from the transcript of the B-cell integration cluster (BIC) gene, also known as the MIR155 host gene (MIR155HG) [17]. The *BIC* gene consists of three exons separated by long (7.6 and 4kb) introns and the stem-loop pre-miR-155 sequence is located in the third exon [18]. MiR-155 is crucial for B-cell development and regulation of the immune response [9,19,20]. High miR-155 levels are observed in many types of cancer, including B-cell malignancies like Hodgkin, primary mediastinal and diffuse large B-cell lymphomas [21,22]. In contrast, very low levels of miR-155 were observed in B cell-derived Burkitt lymphoma [23]. Eis et al. showed that unspliced *BIC* is located in the nucleus, whereas spliced *BIC* is located mainly in the cytoplasm in two B-cell lymphoma cell lines that both show high miR-155 levels [24]. RNA *in situ* hybridization in primary cases of Hodgkin lymphoma and non-Hodgkin lymphomas with high miR-155 levels revealed a strong nuclear staining of *BIC* and no staining in the cytoplasm [21,23].

In this study, we investigated processing of exonic miRNAs, with a main focus on miR-155. We determined the levels of endogenous unspliced and spliced *BIC*, pri-miR-22 and pri-miR-146a transcripts. We assessed cellular localization of endogenous unspliced and spliced *BIC*, pri-miR-22 and pri-miR-146a and showed that unspliced transcripts are located predominantly in the nucleus while spliced transcripts are partly transported to the cytoplasm. We also showed that the 5’ exonic or intronic flanking sequence of pre-miR-155 does not alter processing efficiency of exogenous *BIC* transcripts and that upon overexpression spliced *BIC* transcripts are efficiently processed to mature miR-155.

## Results

### The unspliced/spliced transcript ratio is miRNA-specific in B-cell lymphoma

**Figure 1:**
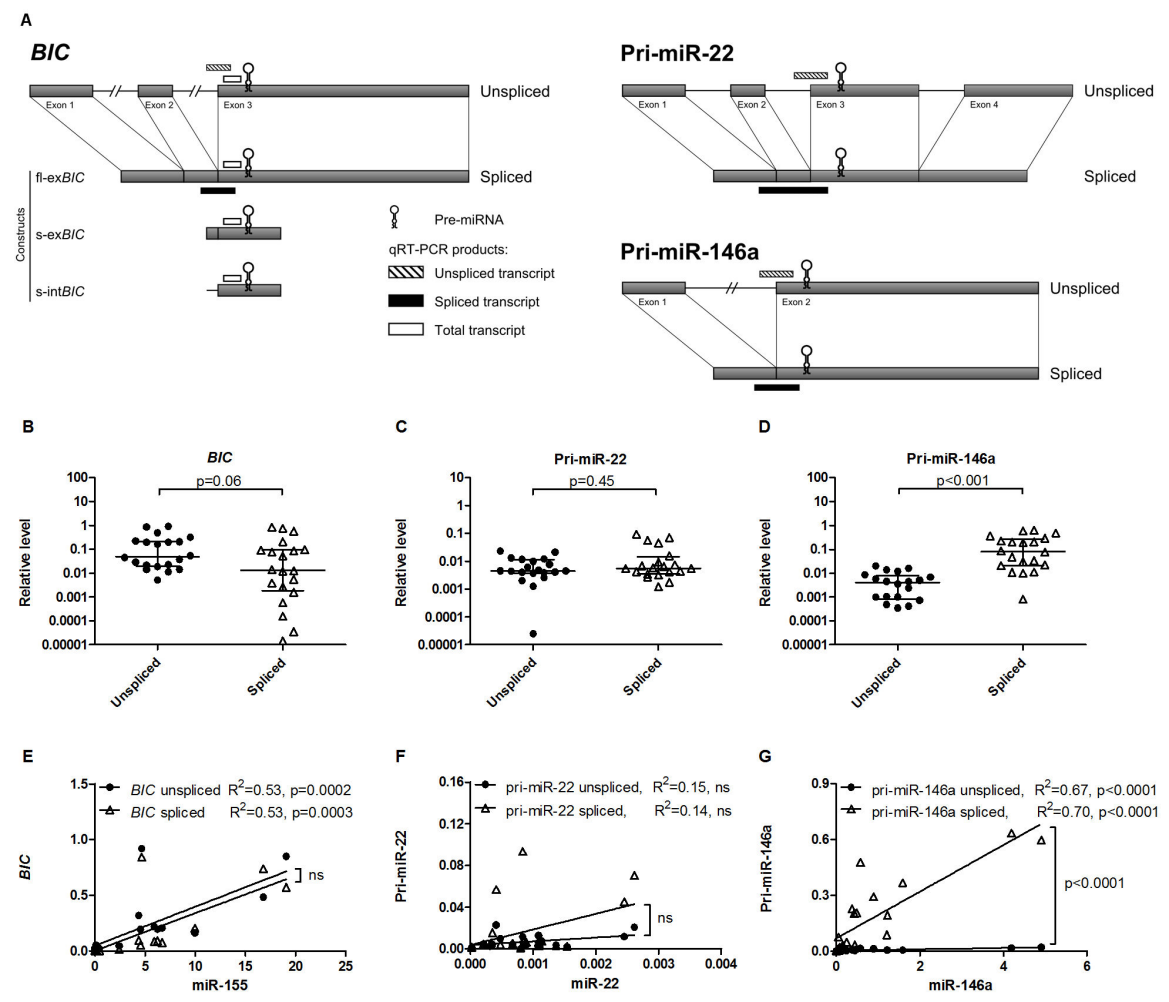
The unspliced/spliced transcript ratio is miRNA-specific in B-cell lymphoma. (A) Schematic overview of the unspliced and spliced *BIC*, pri-miR-22 and pri-miR-146a transcripts and location of the qRT-PCR products specific for unspliced, spliced and total (only for *BIC*) transcripts. Constructs used for miR-155 overexpression are also denoted. Short constructs, s-ex*BIC* and s-int*BIC*, contain the pre-miR-155 sequence and ~150nt 3’ flanking sequence from exon 3 and 5’ flanking sequence derived from exon 2 (s-ex*BIC*) or intron 2 (s-int*BIC*) of *BIC* transcript. The fl-ex*BIC* construct covers the full-length spliced *BIC* transcript. Figures are not drawn to scale. (B) The median endogenous level of unspliced *BIC* transcript (0.049) was 3.7 fold higher than level of spliced *BIC* transcript (0.013). (C) The median endogenous level of unspliced pri-miR-22 (0.004) was similar to level of spliced pri-miR-22 (0.006). (D) The median endogenous unspliced pri-miR-146a level (0.004) was 20.5 fold lower than that of spliced pri-miR-146a (0.081). Levels of *BIC*, pri-miR-22 and pri-miR-146a were assessed in 20 B-cell lymphoma cell lines and normalized to *HPRT* levels. Significance was tested with Mann-Whitney test. (E) Both spliced and unspliced *BIC* showed a similar significant correlation with miR-155 levels. (F) Neither of the two pri-miR-22 transcripts showed a significant correlation with the miR-22 levels. (G) Both unspliced and spliced pri-miR-146a levels significantly correlated with the miR-146a levels. However, the slope of the regression line was significantly steeper for spliced pri-miR-146a.

For exonic miRNAs, such as miR-155, miR-22 and miR-146a, both unspliced and spliced transcripts include the complete stem-loop pre-miRNA sequence and may serve as the primary miRNA transcript. To discriminate between unspliced and spliced transcripts we designed qRT-PCR primer sets specific for unspliced or spliced transcripts as indicated in Figure 1A. We compared the levels of endogenous unspliced and spliced transcripts in twenty B-cell lymphoma cell lines with variable miRNA levels.

Endogenous miR-155 levels were highly variable in B-cell lymphoma cell lines. The difference between the cell line with the lowest (ST486) and the highest (OCI-Ly3) miR-155 level was ~500 fold (Figure S1A). The median level of the unspliced *BIC* transcripts was 3.7 fold higher than that of the spliced *BIC* transcripts (Figure 1B). The levels of endogenous unspliced *BIC* transcripts were higher than the levels of spliced *BIC* transcripts in 17 out of 20 cell lines irrespective of the miR-155 levels (Figure S1B).

MiR-22 levels were low in all B-cell lymphoma cell lines (Figure S1C). Pri-miR-22 has four alternative splice variants. Transcript variant 3 was almost exclusively detected in our panel of B-cell lymphoma cell lines (data not shown). We therefore restricted our subsequent analysis to this splice variant. The medians of endogenous levels of unspliced and spliced pri-miR-22 were similar in 20 analyzed cell lines (Figure 1C). The spliced/unspliced pri-miR-22 transcript ratio varied from 0.2 to 8 (Figure S1D). In SU-DHL-4 cells, the unspliced pri-miR-22 transcript was hardly detected.

MiR-146a levels varied over a 1000-fold range between B-cell lymphoma cell lines (Figure S1E). The levels of spliced pri-miR-146a transcripts were significantly higher than unspliced pri-miR-146a transcripts with a more than 20 fold difference in medians (Figure 1D). The spliced/unspliced pri-miR-146a transcript ratio varied from 14 to 72 fold (Figure S1F). In L540 cells only the spliced pri-miR-146a transcript was present.

Next, we correlated the mature miRNA levels with either unspliced or spliced pri-miRNAs levels. For miR-155, both unspliced and spliced *BIC* transcript levels showed significant correlation with miR-155 levels (R^2^ = 0.53). The difference observed in levels of unspliced and spliced *BIC* transcripts did not result in a significant difference in the slope of the regression lines. For miR-22, no significant correlation was observed for either spliced or unspliced pri-miR-22 transcript levels with mature miR-22 levels. This might be caused by differences in the efficiency of the pri-miR-22 transcript to miR-22 processing, differences in the miR-22 stability, factors regulating the levels of mature miR-22 or limiting amounts of factors involved in the biogenesis of miRNAs. For miR-146a, both unspliced and spliced pri-miR-146a transcript levels significantly correlated with miR-146a (R^2^ = 0.67 and 0.7, respectively). However, the slopes of the curves differed significantly (p<0.0001) due to lower unspliced pri-miR-146a transcript levels.

Thus, for all three exonic miRNAs, both unspliced and spliced primary transcripts are present, albeit at a variable ratio. For *BIC*, the unspliced primary transcript is predominant, for pri-miR-146a the spliced primary transcripts is predominant, whereas for pri-miR-22 the unspliced/spliced transcript ratio varies between cell lines. Levels of the mature miRNAs correlated with levels of both unspliced and spliced pri-miRNA transcripts for miR-155 and miR-146a, but not for miR-22.

### Spliced pri-miRNA transcripts are partly transported to the cytoplasm

**Figure 2:**
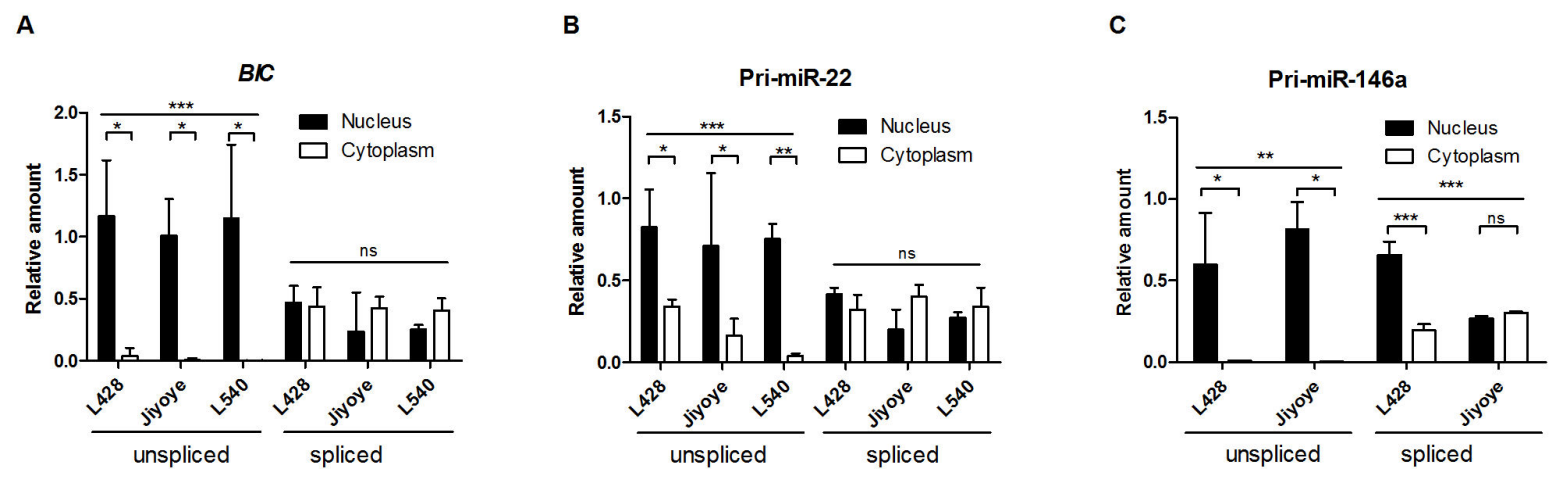
Cellular localization of unspliced and spliced pri-miRNA transcripts. (A) Unspliced *BIC* transcript levels were significantly higher in the nucleus than in the cytoplasm, whereas spliced *BIC* transcripts showed similar levels. (B) Unspliced pri-miR-22 transcripts were more abundant in the nucleus than in the cytoplasm and spliced pri-miR-22 transcript levels were similar. (C) Unspliced pri-miR-146a transcripts were almost exclusively in the nucleus, and spliced pri-miR-146a transcripts were both in the nucleus and in the cytoplasm. Unspliced and spliced pri-miR-146a transcripts were not detectable in L540 cells. Levels of pri-miRNA transcripts in the nucleus or the cytoplasm were calculated relative to their levels in the total fraction and corrected for the amount of RNA per cell. Average of three independent experiments was presented. Significance was calculated using 2-way ANOVA and Bonferroni posttest (* p<0.05, ** p<0.01, *** p<0.001, **** p<0.0001, ns - not significant).

To further examine, whether unspliced or spliced pri-miRNA transcripts are available for miRNA processing, we examined the subcellular localization of unspliced and spliced transcripts of *BIC*, pri-miR-22 and pri-miR-146a by qRT-PCR. We determined the amount of the spliced and unspliced transcripts in the cytoplasm and the nucleus relative to their levels in the total fraction (Figure 2). To asses subcellular localization of unspliced and spliced *BIC* transcripts, we selected cell lines with low (L428), intermediate (Jiyoye) or high (L540) miR-155 levels (Figure S1A). The same cell lines were used for subcellular localization of pri-miR-22 and pri-miR-146a to allow comparison between subcellular distributions of pri-miRNA transcripts. We confirmed purity of the nuclear and cytoplasmic fractions by analyzing relative abundance of tRNA-Lys in the cytoplasm, and *U3* and *Xist* in the nucleus (Figure S2A-C).

Endogenous unspliced *BIC* transcripts were located exclusively in the nuclear fraction in all three cell lines (Figure 2A). Spliced *BIC* transcripts showed similar amounts in the nucleus and in the cytoplasm. Localization of unspliced pri-miR-22 transcripts was predominantly, but not exclusively, nuclear in all three cell lines (Figure 2B). Spliced pri-miR-22 transcripts were present at similar levels in the nucleus and the cytoplasm in all three cell lines. Localization of unspliced pri-miR-146a transcripts was exclusively nuclear for both L428 and Jiyoye cells (Figure 2C). In L540 cells, both mature and primary miR-146a transcripts were hardly detected, so it was not possible to determine the subcellular localization. Spliced pri-miR-146a transcripts were more abundant in the nucleus than in the cytoplasm of L428 cells and in similar amounts in Jiyoye cells.

Thus, part of the spliced *BIC*, pri-miR-22 and pri-miR-146a transcripts are transported to the cytoplasm and as such unavailable for processing. Unspliced transcripts show an almost exclusive nuclear localization for *BIC*, pri-miR-22 and pri-miR-146a. Since the levels of spliced pri-miR-22 in L540 and Jiyoye cells and pri-miR-146a in Jiyoye and L428 were higher than the unspliced transcript levels (Figures S1D and S1F), spliced pri-miR-22 and pri-miR-146a may still be the predominant miRNA substrate.

### Exogenous spliced *BIC* can be processed to miR-155

**Figure 3:**
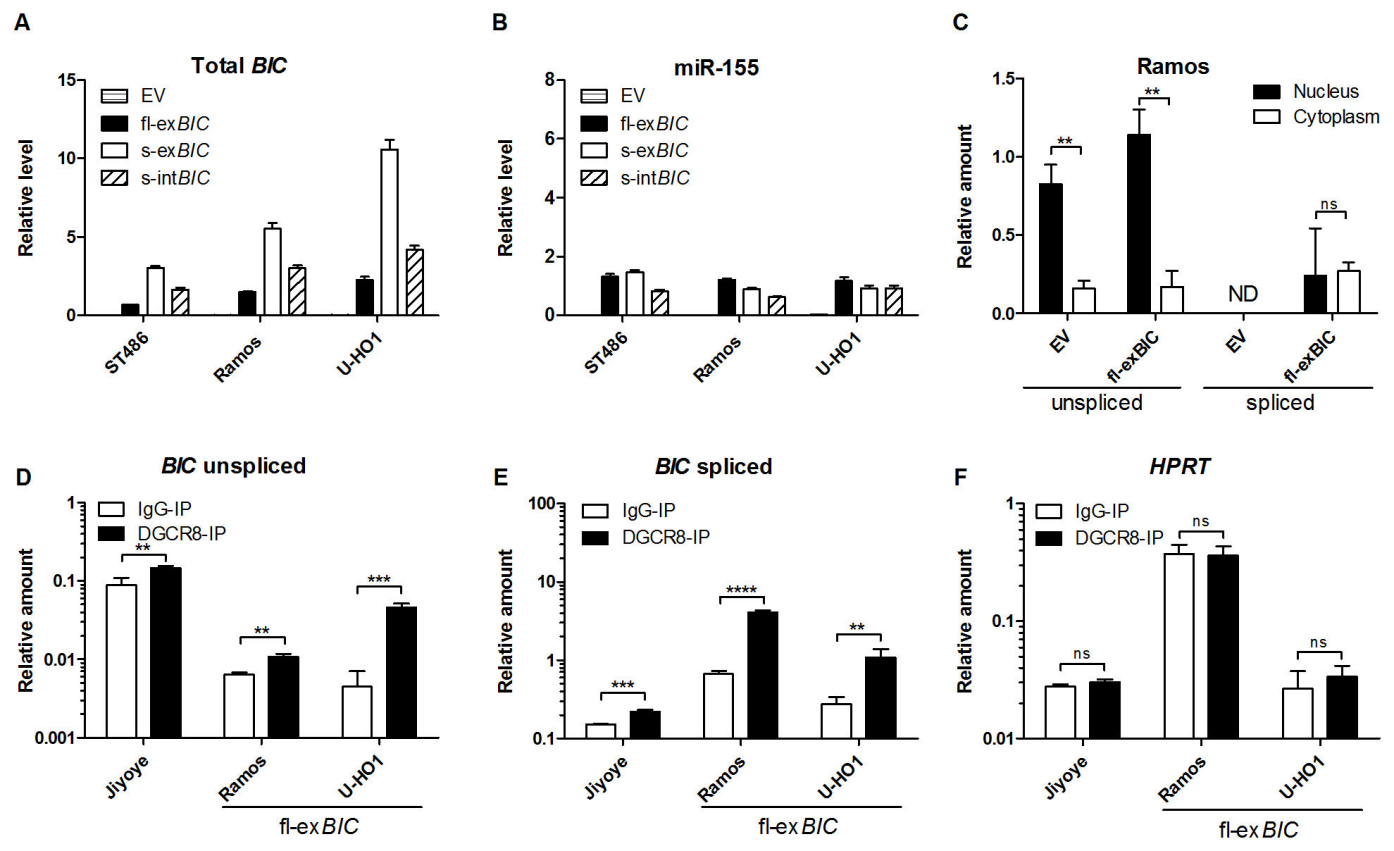
Processing and cellular localization of exogenous *BIC* transcripts. Levels of total *BIC* (A) and miR-155 (B) determined upon overexpression of the three constructs and empty vector (EV) in ST486, Ramos and U-HO1 cells. For the three *BIC* overexpression constructs, the increase in total *BIC* was variable and the highest levels were observed for the short *BIC* transcript containing the exon 2-derived 5’ pre-miR-155 flanking sequence (s-ex*BIC*). However, miR-155 induction was similar for all *BIC* overexpression constructs. (C) The levels of spliced and unspliced *BIC* transcripts in nuclear and cytoplasmic fractions of Ramos EV and Ramos full-length spliced *BIC* (fl-ex*BIC*). For both Ramos EV and fl-ex*BIC*, unspliced *BIC* showed significantly higher (p<0.001) levels in the nucleus than in the cytoplasm. Spliced *BIC* was not detectable (ND) in Ramos EV. Upon overexpression of the fl-ex*BIC* construct, spliced *BIC* transcripts showed similar levels in the cytoplasm and in the nucleus. Transcript levels in the cytoplasm and the nucleus were calculated relative to the total fraction and corrected for the amount of RNA per cell. Average of three independent experiments was presented. P values were determined by 2-way ANOVA and Bonferroni posttest (** p<0.01, ns - not significant). Relative amounts of unspliced (D) and spliced (E) *BIC* transcripts in DGCR8-IP compared to IgG-IP in Jiyoye, Ramos fl-ex*BIC* and U-HO1 fl-ex*BIC* cells. Endogenous unspliced, endogenous spliced and exogenous spliced *BIC* transcripts were significantly enriched in DGCR8-IP compared to IgG-IP. (F) No enrichment of *HPRT* transcript was observed in DGCR8-IP. Amounts were calculated relative to tRNA-Lys levels. P values were determined by Student’s *t*-test (** p<0.01, *** p<0.001, **** p<0.0001, ns - not significant).

For further analysis we focused on miR-155, because previous studies have shown conflicting data concerning *BIC* to miR-155 processing [25,26]. The upstream pre-miR-155 flanking sequence is different in spliced and unspliced *BIC* transcripts. To determine if this upstream sequence affects the processing efficiency, we assessed the levels of miR-155 induction upon overexpression of *BIC* from two short fragments of *BIC* containing the stem-loop region, ~150nt 3’ flanking sequence from exon 3 and ~150nt 5’ flanking sequence derived either from intron 2 (s-int*BIC*) or from exon 2 (s-ex*BIC*) of *BIC* transcript (Figure 1A). In addition, we also overexpressed the full-length spliced *BIC* (fl-ex*BIC*) transcript (Figure 1A). We transduced these three *BIC* constructs into ST486, Ramos and U-HO1, i.e. cells that all have low endogenous miR-155 levels. A high expression of *BIC* was induced using either of the constructs (Figure 3A), albeit at variable levels. Transduction with the short exon spanning *BIC* construct resulted in the highest increase and the full-length spliced *BIC* construct resulted in the lowest increase in total *BIC* transcript levels (Figure 3A). Interestingly, the level of miR-155 induction was similar for all three constructs (Figure 3B), despite the marked differences in total *BIC* levels. These data indicate that the upstream pre-miR-155 flanking sequence does not modify processing efficiency and suggest that at a certain level of primary miRNA transcript, other factors become limiting or regulate the level of mature miR-155.

Next, we determined the subcellular localization of *BIC* transcripts in Ramos cells transduced with fl-ex*BIC* (Figure 3C). Purity of cytoplasmic and nuclear fractions was validated with tRNA-Lys and *U3*, respectively (Fig. S2DE). In empty vector control cells, and in cells with overexpressed fl-ex*BIC*, localization of endogenous unspliced *BIC* transcripts was predominantly nuclear. The endogenous spliced *BIC* transcripts were not detectable in empty vector control cells. The amount of the overexpressed fl-ex*BIC* transcripts was similar in the cytoplasmic and nuclear fractions. Overexpression of fl-ex*BIC* was followed by a strong induction of miR-155 (Figure 3B), indicating that upon overexpression nuclear spliced *BIC* transcripts can also serve as the primary miR-155 transcript.

To show that exogenous spliced *BIC* is processed to miR-155, we determined whether it is a direct target of the Microprocessor complex. We performed immunoprecipitation of DGCR8 [27] in wild-type Jiyoye, Ramos fl-ex*BIC* and U-HO1 fl-ex*BIC* cells (Figure 3D-F and Figure S3A-C). We assessed the relative amounts of unspliced and spliced *BIC* transcripts in the DGCR8 immunoprecipitation fraction (DGCR8-IP) compared to the control IgG-IP. Unspliced *BIC* transcript was significantly more enriched in DGCR8-IP fraction of all three cell lines, albeit at variable levels (Figure 3D). Similarly, the endogenous and exogenous spliced *BIC* transcripts were significantly enriched in DGCR8-IP fraction of Jiyoye, U-HO1 fl-ex*BIC* and Ramos fl-ex*BIC* cells (Figure 3E). The *HPRT* transcript used as negative control was not enriched in the DGCR8-IP fractions (Figure 3F). Enrichment of endogenous and exogenous spliced *BIC* in the DGCR8-IP fractions indicate that the spliced primary transcript can be used for miR-155 processing.

### The unspliced/spliced ratio of *BIC* transcripts changes upon cellular activation

**Figure 4:**
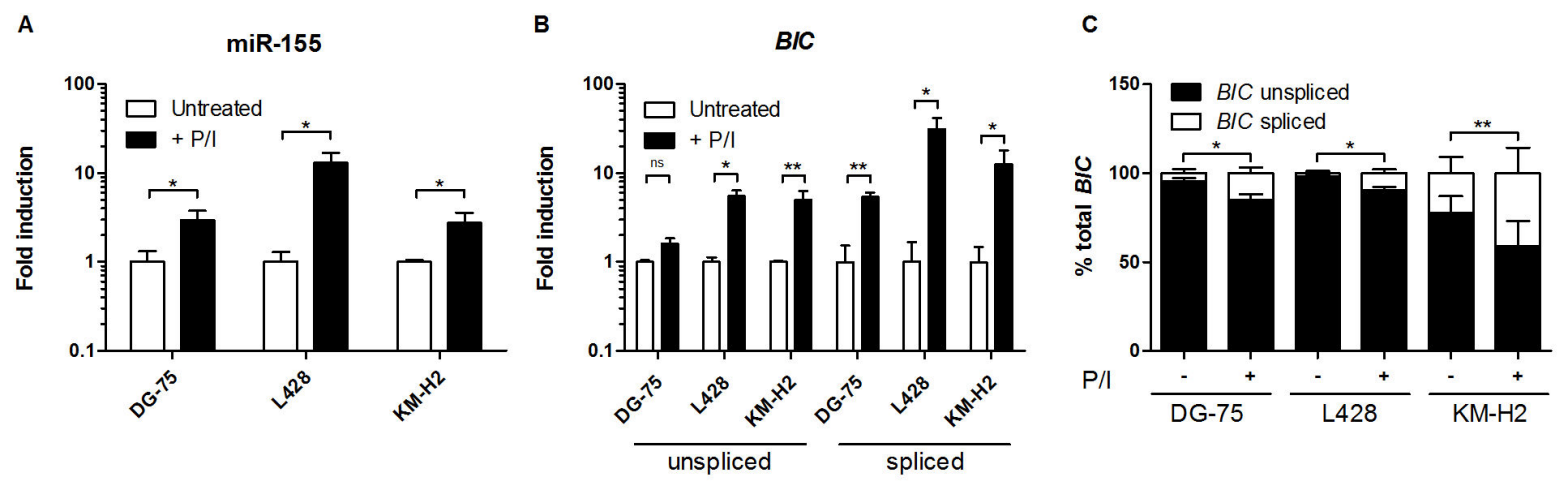
Induction of unspliced and spliced *BIC* transcripts upon cellular activation. Fold induction in miR-155 (A), unspliced *BIC* and spliced *BIC* transcript (B) levels upon PMA/Ionomycin (P/I) treatment. Levels of miR-155 and *BIC* transcripts in untreated cells were averaged and fold induction upon P/I treatment was calculated. In all three cell lines, induction of spliced *BIC* was higher than of unspliced *BIC* transcripts. (C) Unspliced *BIC* transcripts were predominant in both untreated and P/I-treated cells, however unspliced/spliced *BIC* ratio significantly decreased upon P/I treatment. Sum of spliced and unspliced *BIC* transcript levels was set as 100%. Average of three (DG-75 and L428) or four (KM-H2) experiments was presented. P values were determined by a Student’s *t*-test (* p<0.05, ** p<0.01, ns - not significant).

To investigate whether the ratio between unspliced and spliced *BIC* transcripts is altered upon induction *of BIC*, we activated three B-cell lymphoma cell lines using PMA/Ionomycin. Activation of DG-75, L428 and KM-H2 cells resulted in a 3- to 13-fold increase in miR-155 levels (Figure 4A). Induction of unspliced *BIC* transcript levels showed a 1.6 to 5.5 fold increase, whereas spliced *BIC* transcript levels showed a 5.4 to 31 fold increase compared to untreated cells (Figure 4B). Although the unspliced *BIC* transcript remained the predominant transcript, the unspliced/spliced *BIC* transcript ratio significantly changed in favour of the spliced *BIC* transcript (Figure 4C). Thus, both processing of unspliced *BIC* transcript to miR-155 and to spliced *BIC* transcripts are enhanced upon activation-induced expression of *BIC*. In addition, we also assessed induction of mature miR-146a and miR-22 as well as two intronic miRNAs (miR-191, miR-16) and one intergenic miRNA (let-7a). Induction of mature miRNA levels upon PMA/Ionomycin stimulation was observed only for the three exonic miRNAs (Figure S4). Both unspliced and spliced pri-miR-146a and pri-miR-22 transcript levels were induced upon PMA/Ionomycin treatment (Figure S4), however, no difference was observed for the unspliced/spliced transcripts ratios (Figure S4). Thus, the change in unspliced/spliced transcript ratio upon stimulation is specific for the *BIC* transcript.

## Discussion

Exonic miRNAs constitute a small group of the known human miRNAs. The vast majority of exonic miRNAs are located in noncoding RNA genes of which the only known function is being the miRNA host gene. In contrast to intronic miRNAs, processing of the pri-miRNA transcripts of exonic miRNAs to pre-miRNAs interferes with the normal splicing process of the transcript. Exonic miRNAs regulate important physiological pathways, i.e. miR-155 and miR-146a are crucial regulatory components of the immune response, hematopoiesis and carcinogenesis (reviewed in 28,29) and miR-22 plays a role in carcinogenesis [30,31].

In this study, we showed that unspliced pri-miRNA transcripts of exonic miRNAs, i.e. miR-155, miR-22 and miR-146a, are located predominantly in the nucleus. The spliced transcripts are present both in the nucleus and the cytoplasm. Since the first processing step of pri-miRNAs takes place in the nucleus [32,33], both nuclear unspliced and spliced transcripts can serve as pri-miRNA templates. This was supported by presence of both unspliced and spliced *BIC* transcripts in the DGCR8-containing Microprocessor complex. Overexpression constructs containing either the exonic 5’ flanking sequence or the intronic 5’ flanking sequence of pre-miR-155 both resulted in a marked miR-155 induction. Consistent with these findings we also observed a marked induction of miR-155 in cells with overexpressed full-length spliced *BIC* transcripts. These data also indicate that the Microprocessor complex can use unspliced and spliced *BIC* transcripts for processing to pre-miR-155. Since the unspliced *BIC* transcripts are much more abundant and are almost exclusively located in the nucleus, we conclude that unspliced nuclear *BIC* transcripts are the primary template for miR-155 processing in B-cell lymphoma. In contrast to pri-miR-155, the spliced transcripts are the most abundant form of pri-miR-146a and, for part of the cell lines of pri-miR-22. Unspliced pri-miR-146a showed very low levels in all cell lines. This might indicate that the unspliced pri-miR-146a is directly used for processing to pre-miR-146a or for splicing. The level of spliced pri-miR-146a might thus represent the level of pri-miRNA that was not used for miRNA processing. Alternatively, despite the partial cytoplasmic location, the spliced transcripts can still be the most important source for the biogenesis of mature miR-146a. Overexpression of miRNAs from constructs that do not contain introns show very effective processing to mature miRNAs, similar to our results using the *BIC* constructs. This indicates that although splicing enhances processing [12,15], it is not required to allow efficient processing to mature miRNAs. Pawlicki et al. showed that overexpressed pri-miRNAs that are artificially prematurely released from the transcription site accumulate in the nucleoplasm and are not efficiently processed to pre-miRNA [13,34]. These studies implicate that spliced pri-miRNA transcripts may be less efficient templates for the miRNA processing machinery when released from the transcription site. Effective *in vitro* processing of pri-miRNAs using whole-cell extract or immunoprecipitated Microprocessor [6] indicates that presence of the transcript at the transcriptional start site is not required for pre-miRNA release. Although we do not know if spliced exonic pri-miRNA transcripts are released from the transcription start site before miRNA processing, our data clearly show that spliced transcripts can be used for miRNA processing.

We observed that induction of *BIC* using three different constructs was variable. However, the induction of mature miR-155 was strikingly similar in the three cell lines. Thus, induction of higher *BIC* transcripts levels did not result in higher miR-155 levels. This suggests that the miR-155 levels are regulated in these cell lines characterized by very low endogenous miR-155 levels. Notably, the level of miR-155 obtained with these three constructs was still ~10 fold lower than the highest observed endogenous miR-155 levels in OCI-Ly3 cell line. An alternative explanation could be that factors required for miRNA biogenesis become limiting and preclude induction of higher levels.

For *BIC*, we demonstrated that part of the spliced transcripts are exported to the cytoplasm and are thus not available for processing. Similarly, spliced pri-miR-22 and to a lesser degree spliced pri-miR-146a are exported to the cytoplasm. Alteration of the efficiency of splicing and nuclear export of spliced pri-miRNA transcripts may rapidly change the amount of pri-miRNA available for miRNA processing and therefore serve as a mechanism to regulate mature miRNA levels. Consistent with this hypothesis, we observed differences in the ratio of unspliced to spliced *BIC* transcript levels upon PMA/Ionomycin treatment. Cellular conditions and external stimuli may thus affect exonic miRNA levels by inducing changes to the amount of unspliced pri-miRNA used for miRNA processing at the expense of the amount of unspliced transcript available for splicing.

Both unspliced and spliced exonic miRNA transcripts can be used as substrate for miRNA processing. The level and ratio of spliced and unspliced transcripts, their cellular location and the processing efficiency together determine which form is the most likely endogenous pri-miRNA. For exonic miRNA processing studies it is important to assess total transcript levels and not only examine either spliced or unspliced transcripts. Conflicting data as presented in the current literature concerning the processing efficiency of *BIC* may, at least partially, be explained by differences in the analyzed transcripts [25,26].

Many proteins were reported to inhibit or promote miRNA processing by binding to stem-loop of pri-miRNA and/or pre-miRNA (reviewed in 3). These proteins have been identified to regulate both intronic and exonic miRNA processing. KH-type splicing regulatory protein (KSRP) was shown to enhance miR-155 processing in mouse activated macrophages by binding to the terminal loop of both *BIC* transcript and pre-miR-155 [35]. Moreover, monocyte chemoattractant protein [MCP]-1-induced protein 1 (MCPIP1) was shown to suppress miRNA processing of a panel of miRNAs, including miR-155 and miR-146a, by induction of pre-miRNA terminal loops cleavage [36]. Some of these proteins, like KSRP or hnRNPA1, regulate both miRNA and mRNA processing [37-39]. These, and possibly other, regulatory proteins may thus regulate exonic miRNA levels by promoting either pre-miRNA cleavage or splicing of exonic pri-miRNA transcripts.

**Figure 5:**
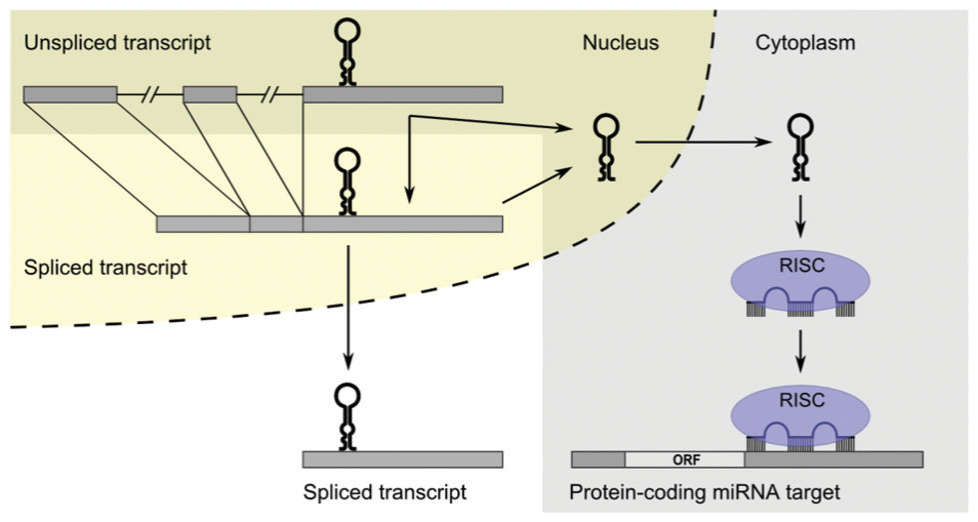
Model of processing of exonic primary miRNA transcripts. The classical miRNA processing and functioning pathway (indicated in dark) shows that the predominant pri-miRNA transcript, i.e. the unspliced nuclear transcript, is processed to pre-miRNA. Pre-miRNA is transported to the cytoplasm and further processed to mature miRNA. Mature miRNA guides the RNA-induced silencing complex (RISC) to protein-coding miRNA targets to inhibit their expression. Unspliced transcripts can also be spliced before being processed to pre-miRNA. Alternatively, the spliced transcript can also be transported to the cytoplasm. This may represent a mechanism to prevent processing to pre-miRNA.

It is unclear whether cytoplasmic spliced pri-miRNA transcripts have a function in the cytoplasm. To date, the only known function of the three noncoding genes studied in this paper is being the host gene for the miRNAs. Splicing of the transcripts and the subsequent transport to the cytoplasm might serve as a mechanism to prevent processing to pre-miRNA (Figure 5). This is supported by the finding that upon inhibition of DGCR8 with shRNA in L1236 cells we saw a marked induction of the spliced *BIC* transcript, which resulted in a change of the unspliced/spliced *BIC* transcript ratio from 2.5 to 0.3 (data not shown). This indicates that when pri-miRNA processing is inhibited, splicing of unspliced *BIC* transcript is enhanced. Another possible role of the cytoplasmic spliced transcripts is that they may function as competing endogenous RNA (ceRNA) transcripts for the mature miRNAs. MiR-155 and miR-155* sequences are highly complementary and the *BIC* transcript is a predicted miR-155 target by the miRanda-mirSVR and PITA algorithms (http://www.microrna.org [40]; http://genie.weizmann.ac.il/pubs/mir07/mir07_prediction.html [41]). Up to date, various transcripts were shown to function as ceRNA, e.g. protein-coding transcripts, pseudogenes, and long noncoding RNAs [42-44]. Spliced pri-miRNA transcripts of exonic miRNAs could prevent binding of the mature miRNA to their endogenous protein-coding target genes and thereby prevent efficient knock down of the target proteins. This would be a novel mechanism, by which cytoplasmic pri-miRNA transcripts function in a negative feedback-back loop to regulate miRNA function. Another possibility is that pri-miRNAs are processed in the cytoplasm, similar to Drosha-mediated processing of viral pri-miRNAs that was shown to take place in the cytoplasm [45]. In these cells relocalization of Drosha to the cytoplasm and the subsequent pri-miRNA processing was triggered by viral infection. This implicates that Drosha relocalization might potentially also occur in normal cells under certain conditions.

In conclusion, we showed that unspliced *BIC*, pri-miR-146a and pri-miR-22 transcripts were predominantly localized in the nucleus, although spliced transcripts are more abundant in some cases. We also showed that spliced *BIC*, pri-miR-146a and pri-miR-22 transcripts are partly localized in the cytoplasm and thus not fully available for processing to the mature miRNAs. Pre-miRNAs and spliced transcripts appear to be two mutually exclusive products of unspliced pri-miRNA transcripts of exonic miRNA. Splicing and transport to the cytoplasm may represent a novel mechanism to regulate cellular exonic miRNA levels and function.

## Materials and Methods

### Cell lines and treatment

Burkitt lymphoma cell lines (Ramos, DG-75, ST486, NAMALWA, Raji, Jiyoye) were purchased from ATCC (ST486) and DSMZ (other cell lines). Diffuse large B-cell lymphoma cell lines (OCI-Ly3, SU-DHL-4, SU-DHL-6, VER) were a kind gift of A. Rosenwald (University of Würzburg, Germany) (OCI-Ly3) [46,47], A. Epstein (UCLA, CA) (SU-DHL-4 and SU-DHL-6) [46,47] or were established in our laboratory (VER) [48]. Hodgkin lymphoma cell lines (L540, L591, L1236, DEV, KM-H2, HDLM-2, L428, U-HO1) were purchased from DSMZ (L540, KM-H2), were a kind gift of V. Diehl (University of Cologne, Germany) (L591 [49], L1236 [50], HDLM-2 [49], L428 [51,52]) and P. Möller (University of Ulm, Germany) (U-HO1) [53] or were established in our laboratory (DEV) [54]. Primary mediastinal B-cell lymphoma cell lines (KARPAS-1106P, MEDB-1) [55] were a kind gift of M. Dyer (University of Leicester, UK). Cell lines were cultured at 37°C under an atmosphere containing 5% CO_2_ in Iscove's Modified Dulbecco's Medium (OCI-Ly3) or RPMI-1640 (other cell lines) medium (Cambrex Biosciences, Walkersville, USA) supplemented with ultraglutamine (2 mM), penicillin (100 U/ml), streptomycin (0.1 mg/ml; Cambrex Biosciences), and 5% (L428), 20% (DEV, ST486, OCI-Ly3) or 10% (other cell lines) fetal calf serum (Cambrex Biosciences). DG-75, L428 and KM-H2 cells were treated for 24h with Phorbol 12-myristate 13-acetate (PMA)/Ionomycin (both Sigma-Aldrich, Saint Louis, MO) as previously described [25]. The PMA/Ionomycin treatment was performed in triplicate (DG-75 and L428) or quadruplicate (KM-H2).

### *BIC*/miR-155 constructs

The pcDNA3.1(+) plasmid containing full-length spliced *BIC* (fl-ex*BIC*) was described previously [25]. The MXW-PGK-IRES-GFP vector was a kind gift from C-Z. Chen (Stanford University, CA). The full-length spliced fl-ex*BIC* insert was subcloned from the pcDNA3.1(+) vector to the MXW-PGK-IRES-GFP vector using PmeI (pcDNA3.1(+) vector) and HpaI (MXW-PGK-IRES-GFP vector) restriction enzymes. The miR-155 stem-loop and ~150nt flanking sequences were amplified from genomic DNA (s-int*BIC*) or cDNA (s-ex*BIC*) using Taq polymerase. Primer sequences used for PCR were as follows, 5’-TGTCACCTCCAGCTTTATAACC-3’ (forward, s-int*BIC*), 5’-AACCTACCAGAGACCTTACC-3’ (forward, s-ex*BIC*), 5'-GGCTTTATCATTTTTCAATCT-3 (reverse, s-int*BIC* and s-ex*BIC*). An XhoI restriction site was added to the forward and an EcoRI site to the reverse primer to allow efficient cloning. PCR products were cloned to the retroviral MXW-PGK-IRES-GFP vector using standard laboratory procedures. The inserts were sequenced to confirm the correct sequences.

### Retroviral transduction

To generate retroviral particles, Phoenix-Ampho packaging cells [56] were CaPO_4_ transfected with 37,5μg of MXW-PGK-IRES-GFP constructs (empty vector or vector containing one of the *BIC* constructs) in T75 flasks. Viral particles were harvested after two days and concentrated with Retro-X concentrator (Clontech, Saint-Germain-en-Laye, France) according to the manufacturer’s protocol. Target cells were transduced with the virus by spinning at 2,000rpm for 2hrs. Cells transduced with retroviral vectors were sorted for GFP using MoFlo sorter (Dako cytomation).

### RNA isolation from total, nuclear and cytoplasmic fractions

Nuclear and cytoplasmic fractions were separated by adding 200µl of lysis buffer (140mM NaCl, 1.5mM MgCl_2_, 10mM Tris-HCl pH8.0, 1mM DTT, 0.5% Nonidet P-40) to pellets of ~4 million cells, followed by 5min incubation on ice and centrifugation for 3min at 4°C and 100xg. The supernatant was harvested as the cytoplasmic fraction. The pellet containing the nuclei was washed twice with lysis buffer. 1ml of Qiazol (Qiagen, Carlsbad, USA) was added to the ~200µl of cytoplasmic fraction, to the nuclear pellet and to the total cell pellet.

### Quantitative RT-PCR

Total RNA was isolated using Trizol (Invitrogen, Carlsbad, USA) according to the manufacturer’s protocol for the cell lines. RNA samples were treated with DNase (Ambion, Foster City, CA). The RNA concentration was measured with a NanoDrop^TM^ 1000 Spectrophotometer (Thermo Fisher Scientific Inc., Waltham, USA) and RNA integrity was evaluated by 1% agarose electrophoresis. cDNA was synthesized using 500ng input RNA, SuperScript II and random primers according to the manufacturers protocol (Invitrogen). The qPCR reaction contained SYBRgreen mix (Applied Biosystems, Foster City, USA), 300nM primers, and 1ng of cDNA in a total volume of 10µl. Levels of spliced, unspliced and total *BIC* as well as spliced and unspliced pri-miR-22, pri-miR-146a were normalized to *HPRT*. For cytoplasmic, nuclear and total fractions, qRT-PCR was performed as described above with slight modifications. We used the miRNeasy kit (Qiagen) including a DNase-treatment (Qiagen) for RNA isolation. RNA was diluted in the same way for total and cytoplasmic fractions and 5 times less for nuclear fraction to use similar amounts (~500ng) for cDNA synthesis. 1/500 of the cDNA was used for qRT-PCR. C_t_ values of nuclear fractions were corrected for different RNA dilutions. Transcript levels in nuclear and cytoplasmic fraction were calculated relative to total fraction. Purity of nuclear fractions was confirmed with *U3* and *Xist* and of the cytoplasmic fraction with tRNA-Lys. For qRT-PCR the following primer sequences used were: for unspliced *BIC*, 5’- AGCTTTATAACCGCATGTGCATAC-3’ (forward) and 5’- CAGATTTCCCCTTCCTGGTTT-3’ (reverse); for total *BIC*, 5’- AAATTCTTTATGCCTCATCCTCTGA-3’ (forward) and 5’-AGGCAAAAACCCCTATCACGAT-3’ (reverse); for unspliced pri-miR-22, 5’-CTGCTCAGATCTTTCCCATTTTC-3’ (forward) and 5’-CCAGGTGAGGGCGTGAGA-3’ (reversed); for spliced pri-miR-22, 5’-GGGCTGATCACTGAACTCACATT-3’ (forward) and 5’-TGAGGGCGTGAGAGGAACA-3’ (reversed), for unspliced pri-miR-146a, 5’-ATTTACCAGGCTTTTCACTCTTGTATT-3’ (forward) and 5’-GGCTTTTCAGAGATGGTGCAA-3’ (reverse); for spliced pri-miR-146a, 5’-GACAGGAGACAGTAGCACAACGA-3’ (forward) and 5’-CAGCCAGCGAGCTCCTAAAA-3’ (reverse); for *Xist*, 5’-GTCCTTTCTTTTGACCCCAGAA-3’ (forward) and 5’-GAGCCTGGCACTTTTTTTTCC-3’ (reverse); primers for spliced *BIC*, *HPRT*, tRNA-Lys and *U3* were described previously [21,57,58]. Localization of unspliced or spliced transcript-specific qRT-PCR products are indicated in Figure 2A. qRT-PCR for miR-155, miR-22, miR-146a, miR-16, miR-191, let-7a and RNU48 was performed using miRNA qRT-PCR assays (Applied Biosystems, Foster City, USA) as described previously [59]. Reverse transcription (RT) primers specific for a miRNA and RNU48 (control) were multiplexed in 15µl RT reactions containing 1µl of each RT primer. The miRNA levels were normalized to the RNU48 levels. Mean cycle threshold (C_t_) values for all genes were quantified with the SDS software (version 2.1). Relative expression levels were calculated as 2^-∆Ct^.

### DGCR8 immunoprecipitation

Immunoprecipitation of DGCR8-containing Microprocessor complexes was performed as described previously for Ago2-IP with some slight modifications [60]. Briefly, ~30 million cells were lysed with 100 µl of the same lysis buffer as used for the cellular fractionation and sonicated for 5 seconds. Lysate was incubated with protein A Sepharose beads (GE Healthcare) coated with anti-DGCR8 antibody (ab90579, Abcam, Cambridge, UK) at 4°C overnight. Mouse IgG antibody was used as a negative control (Millipore BV, Amsterdam, The Netherlands). After washing the beads, RNA was harvested for qRT-PCR analysis and protein lysates were prepared for Western blot. Western blot for DGCR8 was performed as described previously [60], using a 1:2000 dilution of the DGCR8 antibody at 4°C overnight. Total RNA was isolated with miRNeasy Kit (Qiagen) according to manufacturer’s protocol. RNA from DGCR8-IP and IgG-IP fractions of Jiyoye, U-HO1 fl-ex*BIC* and Ramos fl-ex*BIC* cells was used for qRT-PCR analysis. Levels of unspliced and spliced *BIC* transcripts in DGCR8-IP and IgG-IP fraction were calculated relative to tRNA-Lys and fold increase in the DGCR8-IP fraction was calculated for each sample.

## Supporting Information

Figure S1 **The endogenous levels of mature miRNAs, unspliced and spliced pri-miRNAs of miR-155, miR-22 and miR-146a in B-cell lymphoma.** The levels of miR-155 (A), miR-22 (C) and miR-146a (E) in the cell line panel were sorted from low to high. Unspliced and spliced transcripts levels of *BIC* (B), pri-miR-22 (D) and pri-miR-146a (F) in 20 B-cell lymphoma cell lines. In 17 out of 20 cell lines the levels of unspliced *BIC* transcripts were higher than the levels of spliced *BIC* transcripts. Spliced pri-miR-22 transcript levels were higher than unspliced pri-miR-22 transcript levels in 9, similar in 9 and lower in 2 of the analyzed cell lines. In all tested cell lines, levels of spliced pri-miR-146a were much higher than unspliced pri-miR-146a transcripts. Levels of *BIC*, pri-miR-22 and pri-miR-146a were normalized to HPRT and levels of miR-155, miR-22 and miR-146a were normalized to RNU48.
(TIF)

Figure S2 **Validation of nuclear and cytoplasmic fraction purity.** (A) Cytoplasmic control, tRNA-Lys, was mostly present in the cytoplasmic fraction of L428, L540 and Jiyoye cells. From the two nuclear controls, *U3* and *Xist*, *U3* (B) showed slightly higher level in the nucleus and *Xist* (C) was exclusively nuclear in the two female cell lines, L428 and L540. Similarly, for Ramos EV and Ramos fl-ex*BIC*, tRNA-Lys (D) was more abundant in the cytoplasm and *U3* (E) more abundant in the nucleus. Amount of transcripts was calculated relative to the total fraction and corrected for the amount of RNA per cell. Average of 3 experiments was shown.
(TIF)

Figure S3 **Validation of DGCR8-IP fractions.** (A) Western blot for DGCR8 in Ramos fl-ex*BIC* cells. DGCR8 was pulled down with anti-DGCR8 antibody and not with the non-specific IgG control. (B) Levels of unspliced and spliced *BIC* transcripts in total fractions of Jiyoye, Ramos fl-ex*BIC* and U-HO1 fl-ex*BIC* cells. Levels of exogenous spliced *BIC* transcripts were much higher than endogenous unspliced *BIC* transcripts for Ramos and U-HO1. (C) Relative amounts of unspliced and spliced *BIC* transcripts in DGCR8-IP fractions were similar in Jiyoye cells. In the Ramos and U-HO1 cell lines, exogenous spliced *BIC* transcripts were much more abundant in the DGCR8-IP fraction than unspliced *BIC* transcripts.
(TIF)

Figure S4 **Induction of miR-22 and miR-146a upon cellular activation.** Fold induction in levels of miR-22 (A), unspliced and spliced pri-miR-22 (B) in cells treated with PMA/Ionomycin (P/I). Levels of miR-22 were increased in 2 out of 3 cell lines. Levels of unspliced and spliced pri-miR-22 were increased in all tested cell lines. (C) No difference in pri-miR-22 unspliced/spliced ratio was observed upon P/I treatment. Mature miR-146a (D), unspliced and spliced pri-miR-146a (E) transcript levels were also higher P/I-treated cells. (F) Pri-miR-146a unspliced/spliced ratio was not altered upon cellular activation. No differences in levels of intronic miRNAs, miR-16 (G) and miR-191 (H), or intergenic let-7a (I) were observed. Average of 3 experiments was presented. Student’s *t*-test was used to determine p values (*p<0.05, **p<0.01, ***p<0.001, ns - not significant).
(TIF)

## References

[1] Bartel DP (2004) MicroRNAs: Genomics, biogenesis, mechanism, and function. Cell 116: 281-297. doi:10.1016/S0092-8674(04)00045-5. PubMed: 14744438. 14744438 10.1016/s0092-8674(04)00045-5

[2] Calin GA, Sevignani C, Dumitru CD, Hyslop T, Noch E et al. (2004) Human microRNA genes are frequently located at fragile sites and genomic regions involved in cancers. Proc Natl Acad Sci U S A 101: 2999-3004. doi:10.1073/pnas.0307323101. PubMed: 14973191. 14973191 10.1073/pnas.0307323101 PMC365734

[3] Slezak-Prochazka I, Durmus S, Kroesen BJ, van den Berg A (2010) MicroRNAs, macrocontrol: Regulation of miRNA processing. RNA 16: 1087-1095. doi:10.1261/rna.1804410. PubMed: 20423980. 20423980 10.1261/rna.1804410 PMC2874160

[4] Denli AM, Tops BB, Plasterk RH, Ketting RF, Hannon GJ (2004) Processing of primary microRNAs by the microprocessor complex. Nature 432: 231-235. doi:10.1038/nature03049. PubMed: 15531879. 15531879 10.1038/nature03049

[5] Gregory RI, Yan KP, Amuthan G, Chendrimada T, Doratotaj B et al. (2004) The microprocessor complex mediates the genesis of microRNAs. Nature 432: 235-240. doi:10.1038/nature03120. PubMed: 15531877. 15531877 10.1038/nature03120

[6] Han J, Lee Y, Yeom KH, Kim YK, Jin H et al. (2004) The drosha-DGCR8 complex in primary microRNA processing. Genes Dev 18: 3016-3027. doi:10.1101/gad.1262504. PubMed: 15574589. 15574589 10.1101/gad.1262504 PMC535913

[7] Landthaler M, Yalcin A, Tuschl T (2004) The human DiGeorge syndrome critical region gene 8 and its D. melanogaster homolog are required for miRNA biogenesis. Curr Biol 14: 2162-2167. doi:10.1016/j.cub.2004.11.001. PubMed: 15589161. 15589161 10.1016/j.cub.2004.11.001

[8] Kim VN, Nam JW (2006) Genomics of microRNA. Trends Genet 22: 165-173. doi:10.1016/j.tig.2006.01.003. PubMed: 16446010. 16446010 10.1016/j.tig.2006.01.003

[9] Rodriguez A, Vigorito E, Clare S, Warren MV, Couttet P et al. (2007) Requirement of bic/microRNA-155 for normal immune function. Science 316: 608-611. doi:10.1126/science.1139253. PubMed: 17463290. 17463290 10.1126/science.1139253 PMC2610435

[10] Kim YK, Kim VN (2007) Processing of intronic microRNAs. EMBO J 26: 775-783. doi:10.1038/sj.emboj.7601512. PubMed: 17255951. 17255951 10.1038/sj.emboj.7601512 PMC1794378

[11] Saini HK, Enright AJ, Griffiths-Jones S (2008) Annotation of mammalian primary microRNAs. BMC Genomics 9: 564. doi:10.1186/1471-2164-9-564. PubMed: 19038026. 19038026 10.1186/1471-2164-9-564 PMC2632650

[12] Morlando M, Ballarino M, Gromak N, Pagano F, Bozzoni I et al. (2008) Primary microRNA transcripts are processed co-transcriptionally. Nat Struct Mol Biol 15: 902-909. doi:10.1038/nsmb.1475. PubMed: 19172742. 19172742 10.1038/nsmb.1475 PMC6952270

[13] Pawlicki JM, Steitz JA (2008) Primary microRNA transcript retention at sites of transcription leads to enhanced microRNA production. J Cell Biol 182: 61-76. doi:10.1083/jcb.200803111. PubMed: 18625843. 18625843 10.1083/jcb.200803111 PMC2447899

[14] Ballarino M, Pagano F, Girardi E, Morlando M, Cacchiarelli D et al. (2009) Coupled RNA processing and transcription of intergenic primary microRNAs. Mol Cell Biol 29: 5632-5638. doi:10.1128/MCB.00664-09. PubMed: 19667074. 19667074 10.1128/MCB.00664-09 PMC2756881

[15] Kataoka N, Fujita M, Ohno M (2009) Functional association of the microprocessor complex with the spliceosome. Mol Cell Biol 29: 3243-3254. doi:10.1128/MCB.00360-09. PubMed: 19349299. 19349299 10.1128/MCB.00360-09 PMC2698730

[16] Janas MM, Khaled M, Schubert S, Bernstein JG, Golan D et al. (2011) Feed-forward microprocessing and splicing activities at a microRNA-containing intron. PLOS Genet 7: e1002330. 22028668 10.1371/journal.pgen.1002330 PMC3197686

[17] Lagos-Quintana M, Rauhut R, Yalcin A, Meyer J, Lendeckel W et al. (2002) Identification of tissue-specific microRNAs from mouse. Curr Biol 12: 735-739. doi:10.1016/S0960-9822(02)00809-6. PubMed: 12007417. 12007417 10.1016/s0960-9822(02)00809-6

[18] Tam W (2001) Identification and characterization of human BIC, a gene on chromosome 21 that encodes a noncoding RNA. Gene 274: 157-167. doi:10.1016/S0378-1119(01)00612-6. PubMed: 11675008. 11675008 10.1016/s0378-1119(01)00612-6

[19] Thai TH, Calado DP, Casola S, Ansel KM, Xiao C et al. (2007) Regulation of the germinal center response by microRNA-155. Science 316: 604-608. doi:10.1126/science.1141229. PubMed: 17463289. 17463289 10.1126/science.1141229

[20] Vigorito E, Perks KL, Abreu-Goodger C, Bunting S, Xiang Z et al. (2007) microRNA-155 regulates the generation of immunoglobulin class-switched plasma cells. Immunity 27: 847-859. doi:10.1016/j.immuni.2007.10.009. PubMed: 18055230. 18055230 10.1016/j.immuni.2007.10.009 PMC4135426

[21] van den Berg A, Kroesen BJ, Kooistra K, de Jong D, Briggs J et al. (2003) High expression of B-cell receptor inducible gene BIC in all subtypes of hodgkin lymphoma. Genes Chromosomes Cancer 37: 20-28. doi:10.1002/gcc.10186. PubMed: 12661002. 12661002 10.1002/gcc.10186

[22] Kluiver J, Poppema S, de Jong D, Blokzijl T, Harms G et al. (2005) BIC and miR-155 are highly expressed in hodgkin, primary mediastinal and diffuse large B cell lymphomas. J Pathol 207: 243-249. doi:10.1002/path.1825. PubMed: 16041695. 16041695 10.1002/path.1825

[23] Kluiver J, Haralambieva E, de Jong D, Blokzijl T, Jacobs S et al. (2006) Lack of BIC and microRNA miR-155 expression in primary cases of burkitt lymphoma. Genes Chromosomes Cancer 45: 147-153. doi:10.1002/gcc.20273. PubMed: 16235244. 16235244 10.1002/gcc.20273

[24] Eis PS, Tam W, Sun L, Chadburn A, Li Z et al. (2005) Accumulation of miR-155 and BIC RNA in human B cell lymphomas. Proc Natl Acad Sci U S A 102: 3627-3632. doi:10.1073/pnas.0500613102. PubMed: 15738415. 15738415 10.1073/pnas.0500613102 PMC552785

[25] Kluiver J, van den Berg A, de Jong D, Blokzijl T, Harms G et al. (2007) Regulation of pri-microRNA BIC transcription and processing in burkitt lymphoma. Oncogene 26: 3769-3776. doi:10.1038/sj.onc.1210147. PubMed: 17173072. 17173072 10.1038/sj.onc.1210147

[26] Zhang T, Nie K, Tam W (2008) BIC is processed efficiently to microRNA-155 in burkitt lymphoma cells. Leukemia 22: 1795-1797. doi:10.1038/leu.2008.62. PubMed: 18354490. 18354490 10.1038/leu.2008.62

[27] Macias S, Plass M, Stajuda A, Michlewski G, Eyras E et al. (2012) DGCR8 HITS-CLIP reveals novel functions for the microprocessor. Nat Struct Mol Biol 19: 760-766. doi:10.1038/nsmb.2344. PubMed: 22796965. 22796965 10.1038/nsmb.2344 PMC3442229

[28] Tili E, Croce CM, Michaille JJ (2009) miR-155: On the crosstalk between inflammation and cancer. Int Rev Immunol 28: 264-284. doi:10.1080/08830180903093796. PubMed: 19811312. 19811312 10.1080/08830180903093796

[29] Rusca N, Monticelli S (2011) MiR-146a in immunity and disease. Mol. Biol Int: 2011: 437301 10.4061/2011/437301 PMC3200075 22091404

[30] Alvarez-Díaz S, Valle N, Ferrer-Mayorga G, Lombardía L, Herrera M et al. (2012) MicroRNA-22 is induced by vitamin D and contributes to its antiproliferative, antimigratory and gene regulatory effects in colon cancer cells. Hum Mol Genet 21: 2157-2165. doi:10.1093/hmg/dds031. PubMed: 22328083. 22328083 10.1093/hmg/dds031

[31] Xu D, Takeshita F, Hino Y, Fukunaga S, Kudo Y et al. (2011) miR-22 represses cancer progression by inducing cellular senescence. J Cell Biol 193: 409-424. doi:10.1083/jcb.201010100. PubMed: 21502362. 21502362 10.1083/jcb.201010100 PMC3080260

[32] Lee Y, Jeon K, Lee JT, Kim S, Kim VN (2002) MicroRNA maturation: Stepwise processing and subcellular localization. EMBO J 21: 4663-4670. doi:10.1093/emboj/cdf476. PubMed: 12198168. 12198168 10.1093/emboj/cdf476 PMC126204

[33] Yeom KH, Lee Y, Han J, Suh MR, Kim VN (2006) Characterization of DGCR8/Pasha, the essential cofactor for drosha in primary miRNA processing. Nucleic Acids Res 34: 4622-4629. doi:10.1093/nar/gkl458. PubMed: 16963499. 16963499 10.1093/nar/gkl458 PMC1636349

[34] Pawlicki JM, Steitz JA (2009) Subnuclear compartmentalization of transiently expressed polyadenylated pri-microRNAs: Processing at transcription sites or accumulation in SC35 foci. Cell Cycle 8: 345-356. doi:10.4161/cc.8.3.7494. PubMed: 19177009. 19177009 10.4161/cc.8.3.7494 PMC3004524

[35] Ruggiero T, Trabucchi M, De Santa F, Zupo S, Harfe BD et al. (2009) LPS induces KH-type splicing regulatory protein-dependent processing of microRNA-155 precursors in macrophages. FASEB J 23: 2898-2908. doi:10.1096/fj.09-131342. PubMed: 19423639. 19423639 10.1096/fj.09-131342

[36] Suzuki HI, Arase M, Matsuyama H, Choi YL, Ueno T et al. (2011) MCPIP1 ribonuclease antagonizes dicer and terminates microRNA biogenesis through precursor microRNA degradation. Mol Cell 44: 424-436. doi:10.1016/j.molcel.2011.09.012. PubMed: 22055188. 22055188 10.1016/j.molcel.2011.09.012

[37] Trabucchi M, Briata P, Garcia-Mayoral M, Haase AD, Filipowicz W et al. (2009) The RNA-binding protein KSRP promotes the biogenesis of a subset of microRNAs. Nature 459: 1010-1014. doi:10.1038/nature08025. PubMed: 19458619. 19458619 10.1038/nature08025 PMC2768332

[38] Gherzi R, Chen CY, Trabucchi M, Ramos A, Briata P (2010) The role of KSRP in mRNA decay and microRNA precursor maturation. Wiley Interdiscip Rev RNA 1: 230-239. doi:10.1002/wrna.2. PubMed: 21935887. 21935887 10.1002/wrna.2

[39] Michlewski G, Guil S, Semple CA, Cáceres JF (2008) Posttranscriptional regulation of miRNAs harboring conserved terminal loops. Mol Cell 32: 383-393. doi:10.1016/j.molcel.2008.10.013. PubMed: 18995836. 18995836 10.1016/j.molcel.2008.10.013 PMC2631628

[40] Betel D, Koppal A, Agius P, Sander C, Leslie C (2010) Comprehensive modeling of microRNA targets predicts functional non-conserved and non-canonical sites. Genome Biol 11: R90. doi:10.1186/gb-2010-11-8-r90. PubMed: 20799968. 20799968 10.1186/gb-2010-11-8-r90 PMC2945792

[41] Kertesz M, Iovino N, Unnerstall U, Gaul U, Segal E (2007) The role of site accessibility in microRNA target recognition. Nat Genet 39: 1278-1284. doi:10.1038/ng2135. PubMed: 17893677. 17893677 10.1038/ng2135

[42] Poliseno L, Salmena L, Zhang J, Carver B, Haveman WJ et al. (2010) A coding-independent function of gene and pseudogene mRNAs regulates tumour biology. Nature 465: 1033-1038. doi:10.1038/nature09144. PubMed: 20577206. 20577206 10.1038/nature09144 PMC3206313

[43] Tay Y, Kats L, Salmena L, Weiss D, Tan SM et al. (2011) Coding-independent regulation of the tumor suppressor PTEN by competing endogenous mRNAs. Cell 147: 344-357. doi:10.1016/j.cell.2011.09.029. PubMed: 22000013. 22000013 10.1016/j.cell.2011.09.029 PMC3235920

[44] Cesana M, Cacchiarelli D, Legnini I, Santini T, Sthandier O et al. (2011) A long noncoding RNA controls muscle differentiation by functioning as a competing endogenous RNA. Cell 147: 358-369. doi:10.1016/j.cell.2011.09.028. PubMed: 22000014. 22000014 10.1016/j.cell.2011.09.028 PMC3234495

[45] Shapiro JS, Langlois RA, Pham AM, Tenoever BR (2012) Evidence for a cytoplasmic microprocessor of pri-miRNAs. RNA 18: 1338-1346. doi:10.1261/rna.032268.112. PubMed: 22635403. 22635403 10.1261/rna.032268.112 PMC3383965

[46] Epstein AL, Levy R, Kim H, Henle W, Henle G et al. (1978) Biology of the human malignant lymphomas. IV. functional characterization of ten diffuse histiocytic lymphoma cell lines. Cancer 42: 2379-2391.. 214220 10.1002/1097-0142(197811)42:5<2379::aid-cncr2820420539>3.0.co;2-4

[47] Tweeddale ME, Lim B, Jamal N, Robinson J, Zalcberg J et al. (1987) The presence of clonogenic cells in high-grade malignant lymphoma: A prognostic factor. Blood 69: 1307-1314. PubMed: 3567358. 3567358

[48] Atayar C, Poppema S, Blokzijl T, Harms G, Boot M et al. (2005) Expression of the T-cell transcription factors, GATA-3 and T-bet, in the neoplastic cells of hodgkin lymphomas. Am J Pathol 166: 127-134. doi:10.1016/S0002-9440(10)62238-9. PubMed: 15632006. 15632006 10.1016/S0002-9440(10)62238-9 PMC1602286

[49] Diehl V, Pfreundschuh M, Fonatsch C, Stein H, Falk M et al. (1985) Phenotypic and genotypic analysis of hodgkin’s disease derived cell lines: Histopathological and clinical implications. Cancer Surv 4: 399-419. PubMed: 3842319. 3842319

[50] Kanzler H, Hansmann ML, Kapp U, Wolf J, Diehl V et al. (1996) Molecular single cell analysis demonstrates the derivation of a peripheral blood-derived cell line (L1236) from the Hodgkin/Reed-sternberg cells of a hodgkin’s lymphoma patient. Blood 87: 3429-3436. PubMed: 8605361. 8605361

[51] Diehl V, Kirchner HH, Burrichter H, Stein H, Fonatsch C et al. (1982) Characteristics of hodgkin’s disease-derived cell lines. Cancer Treat Rep 66: 615-632. PubMed: 6280862. 6280862

[52] Schaadt M, Diehl V, Stein H, Fonatsch C, Kirchner HH (1980) Two neoplastic cell lines with unique features derived from hodgkin’s disease. Int J Cancer 26: 723-731. doi:10.1002/ijc.2910260605. PubMed: 7216541. 7216541 10.1002/ijc.2910260605

[53] Mader A, Bruderlein S, Wegener S, Melzner I, Popov S et al. (2007) U-HO1, a new cell line derived from a primary refractory classical hodgkin lymphoma. Cytogenet Genome Res 119: 204-210. doi:10.1159/000112062. PubMed: 18253030. 18253030 10.1159/000112062

[54] Atayar C, Kok K, Kluiver J, Bosga A, van den Berg E et al. (2006) BCL6 alternative breakpoint region break and homozygous deletion of 17q24 in the nodular lymphocyte predominance type of hodgkin’s lymphoma-derived cell line DEV. Hum Pathol 37: 675-683. doi:10.1016/j.humpath.2006.01.018. PubMed: 16733207. 16733207 10.1016/j.humpath.2006.01.018

[55] Copie-Bergman C, Boulland ML, Dehoulle C, Möller P, Farcet JP et al. (2003) Interleukin 4-induced gene 1 is activated in primary mediastinal large B-cell lymphoma. Blood 101: 2756-2761. doi:10.1182/blood-2002-07-2215. PubMed: 12446450. 12446450 10.1182/blood-2002-07-2215

[56] Swift S, Lorens J, Achacoso P, Nolan GP (2001) Rapid production of retroviruses for efficient gene delivery to mammalian cells using 293T cell-based systems. Curr Protoc Immunol Chapter 10: Unit 10.17C: Unit 10 17C. PubMed: 18432682 10.1002/0471142735.im1017cs31 18432682

[57] Specht K, Richter T, Müller U, Walch A, Werner M et al. (2001) Quantitative gene expression analysis in microdissected archival formalin-fixed and paraffin-embedded tumor tissue. Am J Pathol 158: 419-429. doi:10.1016/S0002-9440(10)63985-5. PubMed: 11159180. 11159180 10.1016/S0002-9440(10)63985-5 PMC1850313

[58] Taft RJ, Simons C, Nahkuri S, Oey H, Korbie DJ et al. (2010) Nuclear-localized tiny RNAs are associated with transcription initiation and splice sites in metazoans. Nat Struct Mol Biol 17: 1030-1034. doi:10.1038/nsmb.1841. PubMed: 20622877. 20622877 10.1038/nsmb.1841

[59] Gibcus JH, Tan LP, Harms G, Schakel RN, de Jong D et al. (2009) Hodgkin lymphoma cell lines are characterized by a specific miRNA expression profile. Neoplasia 11: 167-176. PubMed: 19177201. 19177201 10.1593/neo.08980 PMC2631141

[60] Tan LP, Seinen E, Duns G, de Jong D, Sibon OC et al. (2009) A high throughput experimental approach to identify miRNA targets in human cells. Nucleic Acids Res 37: e137. doi:10.1093/nar/gkp715. PubMed: 19734348. 19734348 10.1093/nar/gkp715 PMC2777426

