# Supplementary figures and images for: Cellular Localization and Processing of Primary Transcripts of Exonic MicroRNAs
Source: PLoS One. 2013 Sep 20;8(9):e76647. doi: 10.1371/journal.pone.0076647 (PMC3779153; doi:10.1371/journal.pone.0076647)

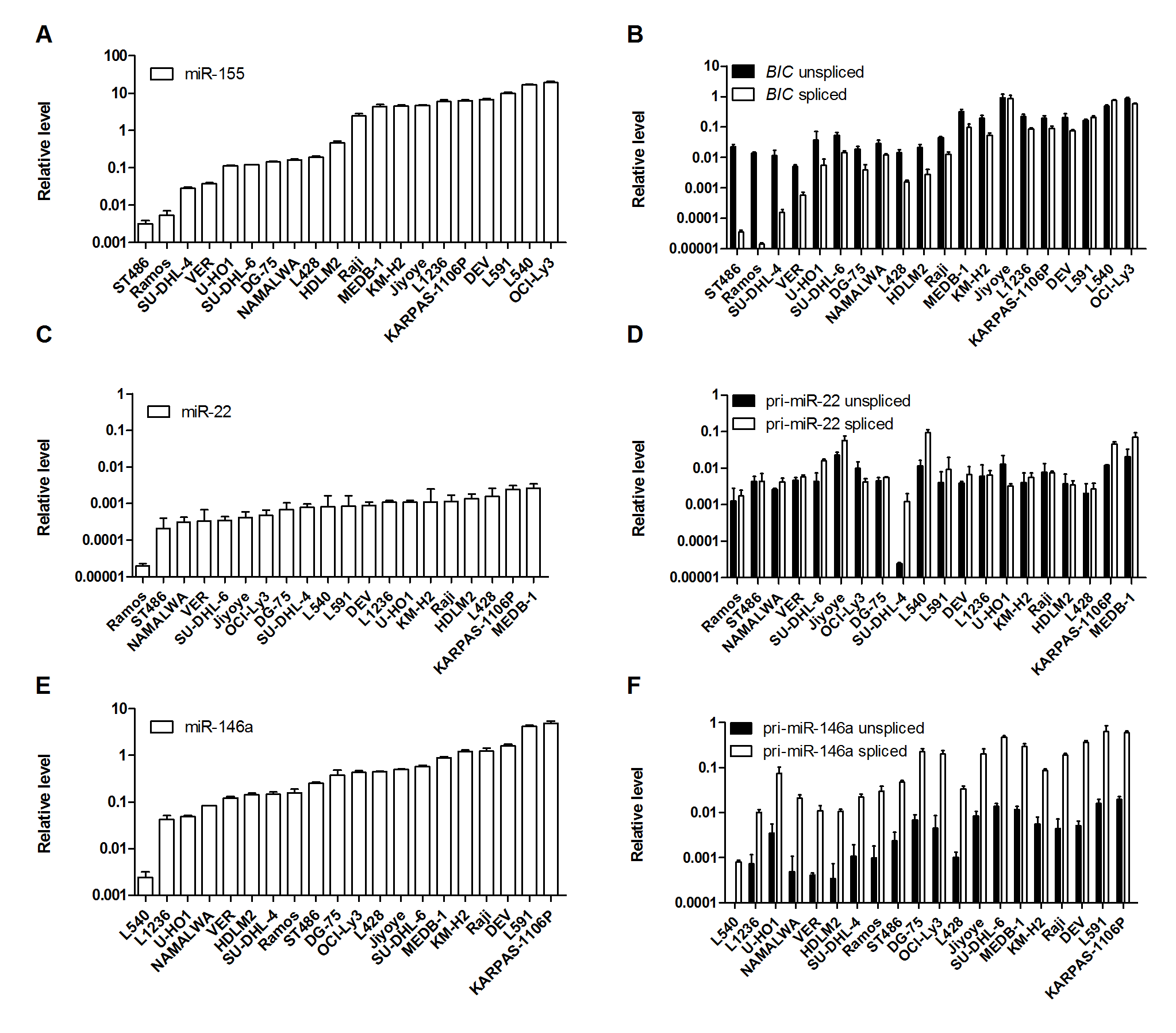

Supplement: Figure S1 — The endogenous levels of mature miRNAs, unspliced and spliced pri-miRNAs of miR-155, miR-22 and miR-146a in B-cell lymphoma. The levels of miR-155 (A), miR-22 (C) and miR-146a (E) in the cell line panel were sorted from low to high. Unspliced and spliced transcripts levels of BIC (B), pri-miR-22 (D) and pri-miR-146a (F) in 20 B-cell lymphoma cell lines. In 17 out of 20 cell lines the levels of unspliced BIC transcripts were higher than the levels of spliced BIC transcripts. Spliced pri-miR-22 transcript levels were higher than unspliced pri-miR-22 transcript levels in 9, similar in 9 and lower in 2 of the analyzed cell lines. In all tested cell lines, levels of spliced pri-miR-146a were much higher than unspliced pri-miR-146a transcripts. Levels of BIC, pri-miR-22 and pri-miR-146a were normalized to HPRT and levels of miR-155, miR-22 and miR-146a were normalized to RNU48. (TIF) [file pone.0076647.s001.tif]

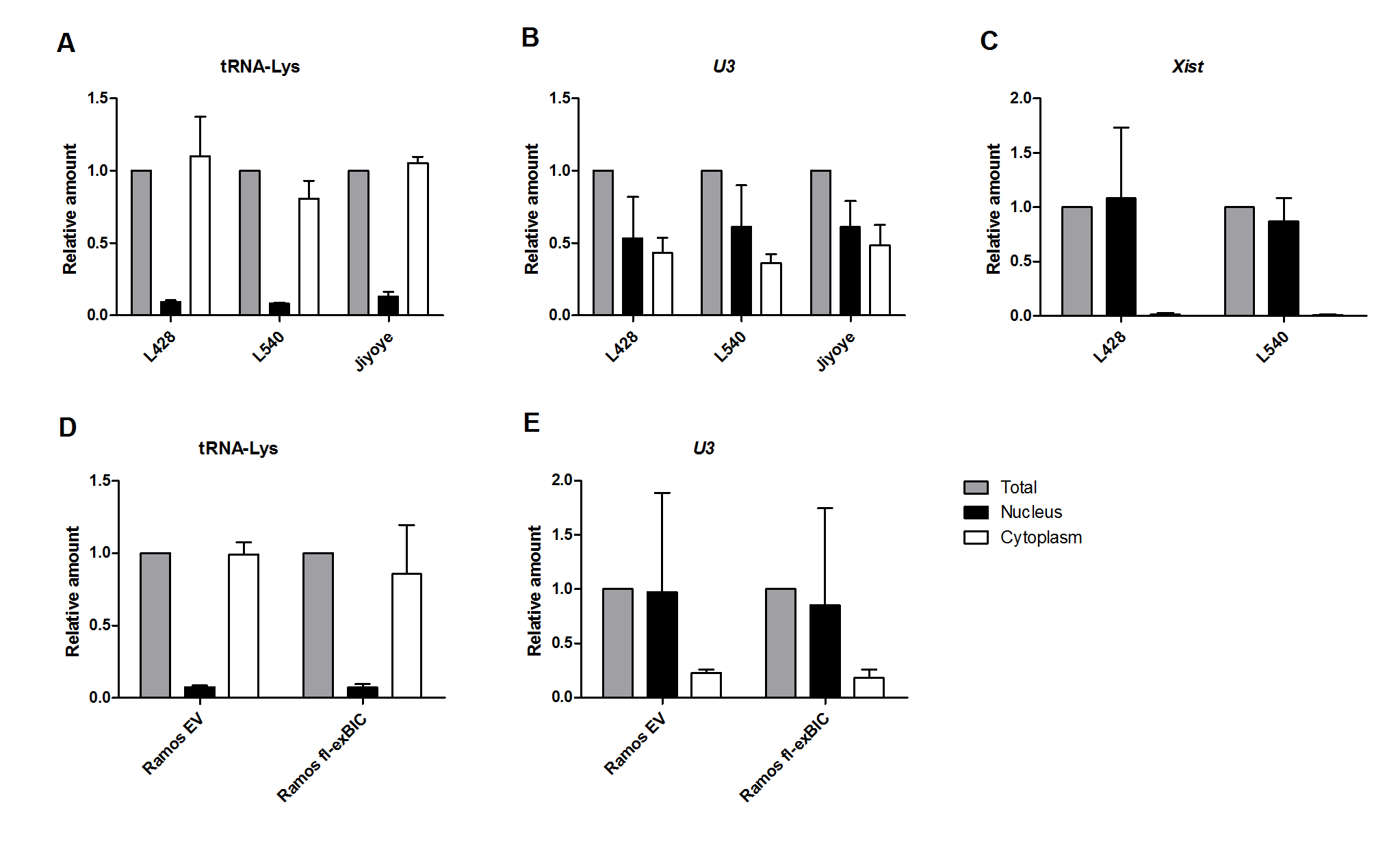

Supplement: Figure S2 — Validation of nuclear and cytoplasmic fraction purity. (A) Cytoplasmic control, tRNA-Lys, was mostly present in the cytoplasmic fraction of L428, L540 and Jiyoye cells. From the two nuclear controls, U3 and Xist, U3 (B) showed slightly higher level in the nucleus and Xist (C) was exclusively nuclear in the two female cell lines, L428 and L540. Similarly, for Ramos EV and Ramos fl-exBIC, tRNA-Lys (D) was more abundant in the cytoplasm and U3 (E) more abundant in the nucleus. Amount of transcripts was calculated relative to the total fraction and corrected for the amount of RNA per cell. Average of 3 experiments was shown. (TIF) [file pone.0076647.s002.tif]

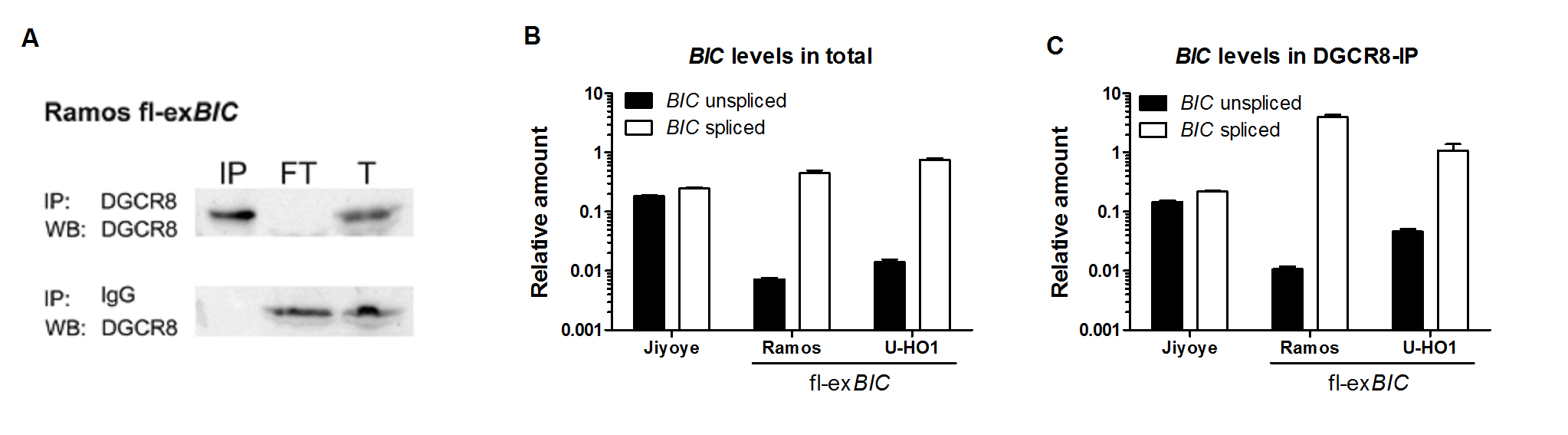

Supplement: Figure S3 — Validation of DGCR8-IP fractions. (A) Western blot for DGCR8 in Ramos fl-exBIC cells. DGCR8 was pulled down with anti-DGCR8 antibody and not with the non-specific IgG control. (B) Levels of unspliced and spliced BIC transcripts in total fractions of Jiyoye, Ramos fl-exBIC and U-HO1 fl-exBIC cells. Levels of exogenous spliced BIC transcripts were much higher than endogenous unspliced BIC transcripts for Ramos and U-HO1. (C) Relative amounts of unspliced and spliced BIC transcripts in DGCR8-IP fractions were similar in Jiyoye cells. In the Ramos and U-HO1 cell lines, exogenous spliced BIC transcripts were much more abundant in the DGCR8-IP fraction than unspliced BIC transcripts. (TIF) [file pone.0076647.s003.tif]

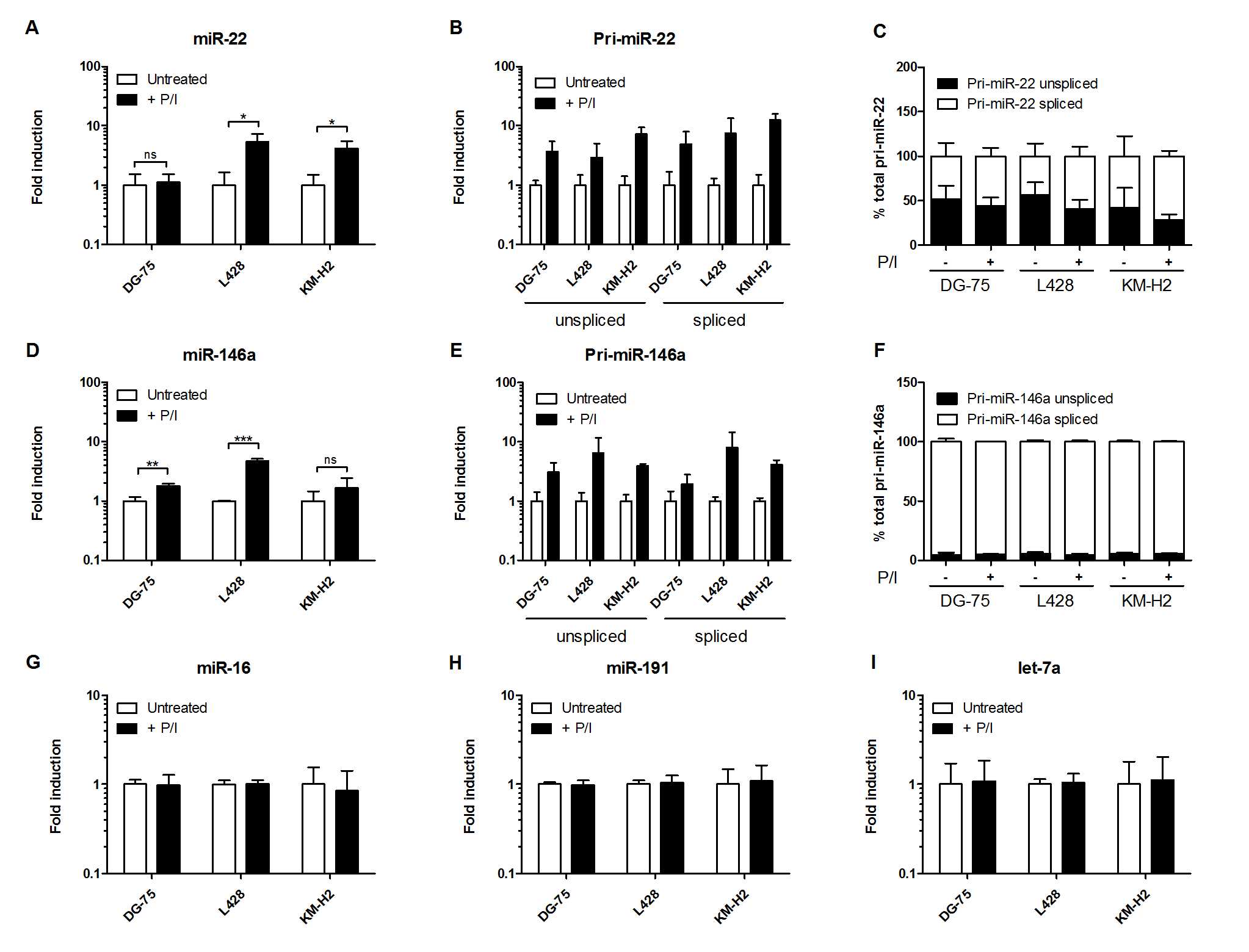

Supplement: Figure S4 — Induction of miR-22 and miR-146a upon cellular activation. Fold induction in levels of miR-22 (A), unspliced and spliced pri-miR-22 (B) in cells treated with PMA/Ionomycin (P/I). Levels of miR-22 were increased in 2 out of 3 cell lines. Levels of unspliced and spliced pri-miR-22 were increased in all tested cell lines. (C) No difference in pri-miR-22 unspliced/spliced ratio was observed upon P/I treatment. Mature miR-146a (D), unspliced and spliced pri-miR-146a (E) transcript levels were also higher P/I-treated cells. (F) Pri-miR-146a unspliced/spliced ratio was not altered upon cellular activation. No differences in levels of intronic miRNAs, miR-16 (G) and miR-191 (H), or intergenic let-7a (I) were observed. Average of 3 experiments was presented. Student’s t-test was used to determine p values (*p<0.05, **p<0.01, ***p<0.001, ns - not significant). (TIF) [file pone.0076647.s004.tif]
